# The perception of individuals in society about protection measures from COVID-19 infection: The example of turkey

**DOI:** 10.1192/j.eurpsy.2021.296

**Published:** 2021-08-13

**Authors:** F. Oflaz, F. Atkan

**Affiliations:** 1 Nursing (psychiatric Nursing), Koç University School of Nursing, Istanbul, Turkey; 2 Nursing (psychiatric Nursing), Koç University Graduate School of Health Sciences, Istanbul, Turkey

**Keywords:** prevention measures, COVID-19, Turkey, perceptions

## Abstract

**Introduction:**

The course of the epidemics such as COVI9 -19 and SARS has taught us that the management of the epidemic depends primarily on people’s adherence to and implementation of the recommended measures.

**Objectives:**

This study aimed to determine the knowledge and opinions of individuals about COVID-19 and transmission methods, sources of information, application status about protection measures and related factors.

**Methods:**

1444 people participated into the digital survey between March 22-April 6, 2020 for this descriptive study. For data collection, a 12-questions questionnaire consisting of questions about the sociodemographic characteristics, information sources they used about the COVID-19, their thoughts the practices to prevent the transmission of COVID-19 was conducted. Using descriptive statistics and comparison tests, individuals’ perceptions about methods of protection from Covid-19 and related variables were investigated.

**Results:**

The participants have had sufficient knowledge about Covid-19 and measures.They were using social media platforms, official web sites and TV news to get information about the COVID-19. The rates of believing and applying measures such as staying distant from people, washing hands, staying at home, avoiding from public transportation, using alcohol disinfectants were quite high. The women, people living in large cities, healthcare workers, regular commuters to work believed in measures more, however, their level of anxiety and seeing themselves and their environment at risk were higher.

**Conclusions:**

Despite all the positive results regarding coronavirus infection and protection measures, the fact that the epidemic is spreading rapidly indicates the need for studies to continuously evaluate what has changed in the process and as time increases.

**Disclosure:**

No significant relationships.

